# Exploring the role of surgical margins and reoperation in basal cell carcinoma recurrence: a study of 3036 cases

**DOI:** 10.1007/s00403-025-04084-3

**Published:** 2025-03-19

**Authors:** Martin Iurilli, Vito Cazzato, Vittorio Ramella, Giovanni Papa

**Affiliations:** Department of Plastic and Reconstructive Surgery, ASUGI, University of Trieste, Trieste, Italy

**Keywords:** Basal cell carcinoma, Skin cancer, Skin biopsy, Surgical therapy, Surgical excision, Excision margins, Reoperation, Recurrence

## Abstract

Introduction: Basal cell carcinoma (BCC) is the most common type of skin cancer globally, with its prevalence increasing due to chronic ultraviolet (UV) radiation exposure. Although surgical excision remains the cornerstone of treatment, achieving optimal outcomes requires a careful balance between complete tumor removal and the preservation of cosmetic appearance. Objective: This study aims to investigate the relationship between excision margins, reoperation rates, and BCC recurrence through a retrospective analysis of 3036 surgical excisions. Methods: Conducted at the Department of Plastic Surgery in Trieste, Italy, this study includes data from 2037 patients treated between 2014 and 2018. Excision procedures adhered to standardized hospital protocols. Tumor characteristics, excision margins, and involved anatomical sites were analyzed. Results: The study demonstrated an equal gender distribution (51.02% women), with the nodular subtype (45.85%) being the most common. The head was the most frequently affected site (54.74%). A complete excision rate of 93.3% was achieved, but incomplete excisions were more common in head and neck locations and certain histological subtypes. Surgical reinterventions were primarily performed for high-risk BCCs, significantly reducing recurrence rates (0.52%). Conclusion: Reoperation, especially for high-risk BCCs, should be prioritized over conservative management to minimize recurrence. Narrow excision margins were associated with higher recurrence rates, highlighting the importance of adequate margin clearance. This study enhances understanding of the complex interplay between excision techniques, reoperation, and long-term outcomes in BCC management.

## Introduction

Basal cell carcinoma (BCC) is the most common form of skin cancer worldwide, accounting for approximately 75% of all cases. It primarily affects fair-skinned individuals, with the incidence varying according to geographical factors, being particularly high among Caucasians in regions closer to the equator [1].

Australia reports the highest incidence rates of BCC, followed by the United States and Europe [2, 3]. In Europe, the average incidence rate in England between 2000 and 2006 was 76.21 per 100,000 person-years [4]. The Netherlands saw a fourfold increase in age-standardized incidence rates for both men and women between 1973 and 2009, reaching 165 and 157 per 100,000 person-years, respectively [5]. Similarly, data from the German cancer registry between 1998 and 2010 revealed a 2.4-fold increase in BCC cases [6]. Despite this rising prevalence, the mortality rates for BCC remain low, with a 5-year absolute survival rate of 87.1% among German patients, consistently exceeding that of the general population by 3–6% [7].

Prolonged ultraviolet (UV) radiation exposure is the primary cause of BCC, with sun-exposed areas of the skin being the most affected. Additional risk factors include genetic predispositions such as Fitzpatrick skin types I-II, certain syndromes like Gorlin-Goltz and Bazex-Dupré-Christol, as well as environmental influences such as X-rays, chemical carcinogens, and immunosuppression. Although BCC is a slow-growing tumor, if left untreated, it can cause significant local tissue damage. Radical surgical excision remains the gold standard for treatment, with the classification of BCCs into low or high-risk categories based on factors such as tumor site, size, and histological subtype.

While 95% of BCCs are low-risk subtypes, easily treated with standard surgical procedures, high-risk BCCs are more prone to recurrence and often require more aggressive management. These high-risk tumors typically involve the H zone of the face (around the nose, eyelids, and ears), aggressive histological subtypes such as basosquamous, sclerosing, infiltrating, or BCC with sarcomatoid differentiation, tumors larger than 2 cm, and cases involving immunosuppressed patients [8].

The management of BCC remains a challenge for plastic surgeons and dermatologic oncologists, requiring a delicate balance between ensuring complete tumor excision and maintaining both functional and aesthetic outcomes. This study aims to provide a comprehensive evaluation of the relationship between excision margins, reoperation rates, and the recurrence of BCC, analyzing 3036 cases from a single-center, retrospective study.

By examining different anatomical sites, the impact of excision margins on recurrence, and the associated patient outcomes, our findings seek to inform clinical decision-making and guide optimal patient care. Precision in surgical intervention for this prevalent skin cancer is paramount, and our study underscores the importance of margin adequacy in minimizing recurrence.

The primary objectives of this study were to determine the correlation between excision margins and the re-intervention rate, as well as the correlation between excision margins and local recurrence. Secondary objectives included examining the relationship between tumor size and re-intervention rates, the correlation between histological subtypes and re-intervention, and the impact of tumor location and subtype on recurrence rates.

## Methods

This retrospective study was conducted at the Department of Plastic, Reconstructive, and Aesthetic Surgery, Trieste - Azienda Sanitaria Universitaria Giuliano Isontina (ASUGI). We enrolled patients who underwent surgical excision for BCC between January 8, 2014, and December 5, 2018. All primary lesions were excised using excisional biopsy, following the ASUGI diagnostic and therapeutic care pathway. The surgeries were performed by five surgeons from the department.

A total of 2037 patients with BCC were included in the analysis. Data were collected from patient clinical and pathological records. Excision procedures adhered to the established care pathway, which included an initial dermatological assessment for diagnosis, followed by consultation with a plastic surgeon for the excision planning. Surgical procedures were performed on an outpatient basis, without preoperative antibiotic prophylaxis. Clinical margins of 4 mm were used for low-risk, well-demarcated tumors smaller than 2 cm, while wider margins of 6–8 mm were applied to high-risk BCCs [9].

For pathological analysis, the “bread loaf technique” was employed for tissue sectioning. Distances between the tumor and the peripheral and deep surgical margins were measured in millimeters. These were categorized into four groups: involved margins, margins less than 1 mm, margins between 1 and 5 mm, and margins greater than 5 mm. Detailed records were kept for all excision margins and their involvement. If positive margins were detected, patients were offered re-excision to ensure complete tumor removal, in line with clinical guidelines [9].

We also recorded additional tumor characteristics, including size, location (such as trunk, back, face, ears, eyelids, and scalp), and histological subtype (nodular, multicentric, sclerosing, basosquamous, infiltrative, superficial, and fibroepithelial).

Statistical analysis was performed using Microsoft Excel and IBM SPSS Statistics 24. The Chi-square test was used for categorical variables to assess relationships and associations, while the Mann-Whitney U test was applied for continuous variables to compare differences between groups. Proportions were compared using the proportion test to assess differences between categorical variables and their distributions.

Patients were followed for an average of 36 months. Recurrence was defined as the appearance of new BCC in the same anatomical site following a previous excision.

The study was conducted in accordance with the ethical standards of the ASUGI ethical committee for clinical practice, and informed consent was obtained from all participants.

## Results

A total of 2037 patients were enrolled, with 3036 primary lesions included in the analysis. The median age at BCC diagnosis was 73.52 years (range: 23.52–100.14) for women and 76.12 years (range: 22.03–97.34) for men. The overall mean age of the patients was 72.6 ± 12.75 years. The mean follow-up time was 36 months (range: 8–60 months).

Several histological subtypes were identified, as listed in Table 1. These subtypes were equally distributed across genders with no statistically significant differences (Chi-Square Test, *p* = 0.393). Among the anatomical sites involved, the head was the most affected, with 1662 cases (54.74%). Within head lesions, the most common sites were the nose (13.31%) and the forehead (9.2%).

**Table 1:** Histological subtypes

Hystological subtypes	Cases (Females)	% (% Females)
Nodular	1392 (710)	45.85 (23.38)
Multicentric	899 (465)	29.61 (15.32)
Sclerosing	369 (194)	12.15 (6.39)
Basosquamous	196 (98)	6.46 (3.22)
Infiltrating	113 (50)	3.72 (1.63)
Superficial	55 (31)	1.81 (1.02)
Fibroepithelial	12 (5)	0.4 (0.17)

Regarding the distance between the tumor and the excision margins, 93.3% (*N* = 2834) of the lesions were excised completely. Of the remaining lesions, 3 (0.1%) involved only the deep margins, 141 (4.6%) involved only the peripheral margins, and 58 (1.9%) had both deep and peripheral margin involvement. The overall incomplete excision rate was 6.7%.

Peripheral margins were the most commonly involved after tumor excision, with 199 cases (6.6%) showing involvement. Deep margin involvement occurred in 61 cases (2%). Table 2 displays the millimeter distances from the tumor to both peripheral and deep margins.

**Table 2:** Margins involvement

**1st surgery peripheral margins**	**Cases**	**%**
involved	199	6.56
< 1 mm	407	13.41
1–5 mm	2213	72.89
≧ 5 mm	217	7.15
**1st surgery deep margins**		
involved	61	2.01
< 1 mm	260	8.56
1–5 mm	1390	45.78
≧ 5 mm	1325	43.64

Reintervention was recommended for all patients with positive peripheral and/or deep margins (6.7%, *N* = 202) and for those whose margins were less than 0.3 mm (3.6%, *N* = 108). A total of 146 patients with involved margins and 61 patients with margins less than 0.3 mm underwent reoperation.

Patients who did not undergo reintervention, due to personal choice or poor clinical condition, were referred for dermatologic follow-up. Of those with involved margins, 27.7% (*N* = 56) chose follow-up, and 85.3% (*N* = 353) of those with margins less than 1 mm chose follow-up. There was one clinical recurrence in the follow-up group, a multicentric BCC on the ear that was not reoperated.

The sites most frequently requiring reintervention were the nose (30%), eyelids (15%), ears (15%), cheeks (15%), and forehead (10%).

Following re-excision (*N* = 207), the results for peripheral margin involvement were as follows: 15 cases (7.25%) had involved margins, 11 cases (5.31%) had margins less than 1 mm from the tumor, 16 cases (7.73%) had margins between 1 and 5 mm, and 165 cases (79.71%) had margins greater than 5 mm. For deep margins, 1 case (0.48%) showed involvement, 1 case (0.48%) had margins less than 1 mm, 12 cases (5.80%) had margins between 1 and 5 mm, and 179 cases (86.47%) had margins greater than 5 mm. For 14 cases (6.77%), the margin data was not recorded.

The histological subtypes most often requiring reintervention were sclerosing (14.09%), basosquamous (10.20%), infiltrating (7.08%), multicentric (6.67%), nodular (4.7%), and superficial (1.19%). This difference was statistically significant (*p* < 0.001, proportion test).

During follow-up, 16 recurrences (0.52%) were observed. Recurrences were more frequent in anatomical areas such as the nose (*N* = 4) and forehead (*N* = 3). Overall, facial sites were the most commonly affected by recurrence.

Lesions that recurred had significantly narrower free margins compared to those that did not recur, with mean margins of 1.22 mm versus 1.89 mm (Mann-Whitney test *p* = 0.002).

The histological subtypes of recurrent BCCs were as follows: 8 cases of nodular, 3 of multicentric, 2 of sclerosing, and 3 of basosquamous. No recurrences occurred in infiltrative, superficial, or fibroepithelial subtypes.

The nose was the most frequent site of recurrence (4 lesions), followed by the forehead (3 lesions). Other sites of recurrence included the trunk (2 lesions), ears (2 lesions), and isolated cases in the lip, neck, eyelids, lower limbs, and face (*p* = 0.07).

## Discussion

In our study, we observed a balanced gender distribution of BCC cases, with a slight preponderance of women (51.02% vs. 48.98%), which aligns with existing literature [10]. This discrepancy, however, did not reach statistical significance (*p* = 0.393), suggesting that gender does not significantly influence the incidence of BCC in our cohort. A higher incidence in elderly patients was observed, with mean ages of 73.52 years for men and 76.12 years for women. These findings are consistent with the broader literature, which underscores aging as a key demographic factor in the development of BCC [11, 12].

The dominance of the nodular subtype (45.85%) in our study is in line with the clinical significance of this BCC variant, which is the most common subtype in most populations. Notably, almost 60% of these cases appeared on the face, particularly the nose and forehead, highlighting the critical role of facial sun exposure in the development of nodular BCC [13, 14]. The distribution of histological subtypes did not vary significantly by sex, reinforcing the notion that these subtypes are equally prevalent across genders in our cohort.

Regarding anatomical distribution, the head emerged as the most affected region, with 54.74% of cases localized to the face, particularly the nose (13.31%) and forehead (9.2%). This finding further supports the well-established link between sun exposure and the higher incidence of BCC in sun-exposed areas, particularly the facial regions. These results emphasize the need for enhanced sun protection measures, especially in high-risk areas like the face.

Our study achieved a high complete excision rate of 93.3%, with an incomplete excision rate of 6.7%, which is slightly below the lower limits of 7–25% reported in the literature [15]. Incomplete excision rates were higher in specific regions, such as the head and neck, and with certain histological subtypes, aligning with findings from other studies that highlight the challenges of excising tumors in these anatomically and cosmetically sensitive areas [16–18].

Factors contributing to incomplete excisions include both tumor location and histological type. For example, tumors in areas such as the nose, eyelids, and nasolabial folds are more likely to be incompletely excised due to the aesthetic concerns of extensive resection. This phenomenon underscores the need for individualized surgical approaches in such anatomically complex regions. In addition, histological subtypes such as sclerosing and infiltrative BCCs are at higher risk of incomplete excision, as they tend to infiltrate deeper into the tissue, making complete removal more challenging [19–21].

In our study, peripheral margin involvement was observed in 6.6% of cases, which is consistent with existing research and highlights the importance of ensuring complete excision beyond the clinically visible tumor boundary [17]. Notably, we found that peripheral margins greater than 5 mm were exceeded in 7.15% of cases, with a significant proportion (72.89%) of excisions falling within the 1–5 mm range. This further emphasizes the delicate balance between achieving clear margins and preserving functional and cosmetic outcomes in anatomically sensitive areas.

Some authors suggest that dermoscopy improves margin delineation for surgical excision of BCC. The identification of nontraditional dermoscopic features, such as pinkish-white areas and short telangiectasias between clinical and dermoscopic margins, helps determine the true boundaries of the tumor, facilitating accurate excision [22].

In terms of clinical practice, our study strongly supports the importance of reintervention in cases of positive or narrow margins, especially in high-risk tumors located on the face or with aggressive subtypes. While some studies advocate for a more conservative “watch and wait” approach in cases with only lateral margin involvement, our results favor a proactive surgical approach to mitigate recurrence risks, in line with recommendations for excising all BCCs with positive margins [23, 24].

Our data also suggest a significant correlation between tumor size and reintervention rates. Tumors requiring reintervention were notably larger than those that were successfully excised on the first attempt, with lesions over 10 mm in diameter showing a significantly higher likelihood of requiring further excision (*p* = 0.0049). This underscores the need for wider clinical margins during the initial excision of larger tumors to reduce the need for subsequent interventions and improve long-term outcomes.

We found that the subtypes most frequently undergoing reintervention were sclerosing (14.09%) and basosquamous (10.20%). These aggressive subtypes warrant more radical excisions with larger clinical margins, particularly in high-risk anatomical regions such as the face.

According to the literature, fully excised tumors have recurrence rates of about 5.9%, while those that are not entirely excised have recurrences in an average of 26.8% of cases [15].

Regarding recurrence, our study reports a low overall recurrence rate of 0.52%, which is promising compared to the recurrence rate of 1% for fully excised tumors reported in the literature [25]. Tumors with closer excision margins had significantly higher recurrence rates, emphasizing the importance of achieving adequate margins during initial excision (*p* = 0.002). Recurrence was more frequent in facial regions, particularly the nose and forehead, areas where aesthetic considerations may lead to more conservative excision techniques. This highlights the challenges of balancing clear margins with cosmetic outcomes in these sensitive areas.

Interestingly, basosquamous carcinoma exhibited a higher recurrence rate (1.6%) compared to other subtypes, which may warrant further investigation into the unique biological characteristics of this subtype and its implications for surgical management.

While our study provides valuable insights into BCC excision, it is essential to acknowledge its limitations, including its retrospective design and the potential variability in surgical techniques across operators. The relatively small number of recurrences may also limit the generalizability of some of our findings. Despite these limitations, the study’s strength lies in its large sample size of 3036 lesions, which provides robust data to guide clinical practice. Future studies could explore additional factors, such as genetic and environmental variables, to further refine BCC treatment strategies and outcomes.

## Conclusion

Surgical excision with clinically clear margins, as recommended by the NCCN guidelines (4 mm for suspected BCCs), is essential to minimize the risk of recurrence and ensure optimal outcomes. Additionally, regular dermatologic follow-up is crucial for the early detection of any recurrences, which can be managed more effectively if identified promptly.

Particular attention must be given to high-risk BCCs, including those located on the face, and those with aggressive histological subtypes, such as sclerosing and basosquamous, or those exceeding 10 mm in size. For these tumors, the widest possible excision is recommended, as they are more prone to recurrence.

Our findings provide significant insights into the complex relationship between excision margins, the need for reoperation, and long-term outcomes in BCC patients. The methodological approach we utilized offers a strong foundation for further studies exploring the intricate balance between excision extent and patient prognosis, guiding clinical decisions and improving patient care in the management of basal cell carcinoma.

## Data Availability

No datasets were generated or analysed during the current study.

## References

[1] Dessinioti C, Antoniou C, Katsambas A, Stratigos AJ Basal cell carcinoma: what’s new under the sun. Photochem Photobiol 2010 May-Jun;86(3):481–491 10.1111/j.1751-1097.2010.00735.x 20550646

[2] Staples MP, Elwood M, Burton RC, Williams JL et al (2006) Non-melanoma skin cancer in Australia: the 2002 National survey and trends since 1985. Med J Aust 184:6e10 16398622 10.5694/j.1326-5377.2006.tb00086.x

[3] Holm AS, Nissen CV, Wulf HC (2016) Basal cell carcinoma is as common as the sum of all other cancers: implications for treat- ment capacity. Acta Derm Venereol 96:505e9 26554445 10.2340/00015555-2282

[4] Lomas A, Leonardi-Bee J, Bath-Hextall F (2012) A systematic review of worldwide incidence of nonmelanoma skin cancer. Br J Dermatol 166(5):1069e80 22251204 10.1111/j.1365-2133.2012.10830.x

[5] Flohil SC, Seubring I, van Rossum MM, Coebergh JW et al (2013) Trends in basal cell carcinoma incidence rates: a 37-year Dutch observational study. J Invest Dermatol 133:913e8 23190883 10.1038/jid.2012.431

[6] Rudolph C, Schnoor M, Eisemann N, Katalinic A (2015) Incidence trends of nonmelanoma skin cancer in Germany from 1998 to 2010. J Dtsch Dermatol Ges 13:788e97 26213814 10.1111/ddg.12690

[7] Eisemann N, Jansen L, Castro FA, Chen T et al (2016) Survival with nonmelanoma skin cancer in Germany. Br J Dermatol 174:778e85 26676514 10.1111/bjd.14352

[8] Peris K, Fargnoli MC, Garbe C, Kaufmann R et al (2019) Diagnosis and treatment of basal cell carcinoma: European consensus-based interdisciplinary guidelines. Eur J Cancer 118:10–34 31288208 10.1016/j.ejca.2019.06.003

[9] Schmults CD, Blitzblau R, Aasi SZ et al (2023) Basal cell skin cancer, version 2.2024, NCCN clinical practice guidelines in oncology. J Natl Compr Canc Netw 21(11):1181–1203 37935106 10.6004/jnccn.2023.0056

[10] Gomes R, Nascimento EF, Araújo FC (2007) Why do men use health services less than women? Explanations by men with low versus higher education. Cad Saude Publica 23:565–574 17334571 10.1590/s0102-311x2007000300015

[11] Chinem VP, Miot HA (2011) Epidemiology of basal cell carcinoma. Bras Dermatol 86:292–305 10.1590/s0365-05962011000200013 21603813

[12] Basset-Seguin N, Herms F (2020) Update in the management of basal cell carcinoma. Acta Derm Venereol 100(11):adv00140 32346750 10.2340/00015555-3495 PMC9189749

[13] Scrivener Y, Grosshans E, Cribier B (2002) Variations of basal cell carcinomas according to gender, age, location and histopathological subtype. Br J Dermatol 147(1):41–47 12100183 10.1046/j.1365-2133.2002.04804.x

[14] Soyer HP, Rigel DS, Wurm EM (2012) In: Dermatology. 3rd edition. Bolognia JL, Jorizzo JL, Schaffer JV, editors. Basal Cell Carcinoma and Squamous Cell Carcinoma. Saunders;; pp. 1773–1793

[15] Codazzi D, Van Der Velden J, Carminati M, Bruschi S et al (2014) Positive compared with negative margins in a single-centre retrospective study on 3957 consecutive excisions of basal cell carcinomas. Associated risk factors and preferred surgical management. J Plast Surg Hand Surg 48:38–43 23731130 10.3109/2000656X.2013.800526

[16] Bogdanov-Berezovsky A, Cohen AD, Glesinger R et al (2004) Risk factors for incomplete excision of basal cell carcinomas. Acta Derm Venereol 84:44–47 15040477 10.1080/00015550310020585

[17] Griffiths RW, Suvarna SK, Stone J (2007) Basal cell carcinoma histological clearance margins. An analysis of 1539 conventionally excised tumours. Wider still and deeper? J Plast Reconstr Aesthet Surg 60:41–47 17126265 10.1016/j.bjps.2006.06.009

[18] Farhi D, Dupin N, Palangié A, Carlotti A et al (2007) Incomplete excision of basal cell carcinoma: rate and associated factors among 362 consecutive cases. Dermatol Surg 33(10):1207–1214 17903153 10.1111/j.1524-4725.2007.33255.x

[19] Nagore E, Grau C, Molinero J et al (2003) Positive margins in basal cell carcinoma: relationship to clinical features and recurrence risk. A retrospective study of 248 patients. J Eur Acad Dermatol Venereol 17:167–170 12705745 10.1046/j.1468-3083.2003.00535.x

[20] Sexton M, Jones DB, Maloney ME (1990) Histologic pattern analysis of basal cell carcinoma: study of a series of 1039 consecutive neo- plasms. J Am Acad Dermatol 23:1118–1126 2273112 10.1016/0190-9622(90)70344-h

[21] Godoy CAP, Neta ALO, Leão SSS, Dantas RL et al Evaluation of surgical margins according to the histological type of basal cell carcinoma. Bras Dermatol 2017 Mar-Apr;92(2):226–230 10.1590/abd1806-4841.20175076 PMC5429110 28538884

[22] Conforti C, Giuffrida R, Zalaudek I, Guarneri F et al (2021) Dermoscopic findings in the presurgical evaluation of basal cell carcinoma. A prospective study. Dermatol Surg 47(2):e37–e41 32804889 10.1097/DSS.0000000000002471

[23] Fernandes JD, de Lorenzo Messina MC, de Almeida Pimentel ER, Castro LG (2008) Presence of residual basal cell carcinoma in re-excised specimens is more probable when deep and lateral margins were positive. J Eur Acad Dermatol Venereol 22:704–706 18384541 10.1111/j.1468-3083.2007.02571.x

[24] Longhi P, Serra MP, Robotti E (2008) Incompletely excised basal cell carcinomas: our guidelines. Onco Targets Ther 1:1–4 21127747 10.2147/ott.s3540 PMC2994206

[25] Griffiths RW, Suvarna SK, Stone J (2005) Do basal cell carcinomas recur after complete conventional surgical excision? Br J Plast Surg 58:795e805 16086990 10.1016/j.bjps.2005.02.010

